# Current Non-Contact Road Surface Condition Detection Schemes and Technical Challenges

**DOI:** 10.3390/s22249583

**Published:** 2022-12-07

**Authors:** Yao Ma, Meizhu Wang, Qi Feng, Zhiping He, Mi Tian

**Affiliations:** 1 Key Laboratory of Space Active Opto-Electronics Technology, Shanghai Institute of Technical Physics, Chinese Academy of Sciences, Shanghai 200083, China; 2 Key Laboratory of Infrared System Detection and Imaging Technology, Shanghai Institute of Technical Physics, Chinese Academy of Sciences, Shanghai 200083, China; 3 School of Information Science and Technology, Shanghai Tech University, Shanghai 201210, China; 4 University of Chinese Academy of Sciences, Beijing 100049, China; 5 Shanghai Pushi Technology Co., Ltd., Shanghai 200083, China

**Keywords:** road surface condition, friction coefficient, autonomous driving, non-contact detection, infrared spectroscopy

## Abstract

Given the continuous improvement in the capabilities of road vehicles to detect obstacles, the road friction coefficient is closely related to vehicular braking control, thus the detection of road surface conditions (RSC), and the level is crucial for driving safety. Non-contact technology for RSC sensing is becoming the main technological and research hotspot for RSC detection because of its fast, non-destructive, efficient, and portable characteristics and attributes. This study started with mapping the relationship between friction coefficients and RSC based on the requirement for autonomous driving. We then compared and analysed the main methods and research application status of non-contact detection schemes. In particular, the use of infrared spectroscopy is expected to be the most approachable technology path to practicality in the field of autonomous driving RSC detection owing to its high accuracy and environmental adaptability properties. We systematically analysed the technical challenges in the practical application of infrared spectroscopy road surface detection, studied the causes, and discussed feasible solutions. Finally, the application prospects and development trends of RSC detection in the fields of automatic driving and exploration robotics are presented and discussed.

## 1. Introduction

With the increase in road mileage and the substantial increase in the number of drivers and vehicles, road traffic safety has attracted increasing attention in the field of driving. The road friction coefficient is closely related to vehicular braking control. The friction coefficient of low-road surfaces introduces high-side-slip risks, because of which brakes become ineffective, and it introduces other problems that lead to traffic accidents; accordingly, its impact on driving safety is considerable. Factors affecting the friction coefficient of vehicles during driving include the vehicle itself, road surface, road-body medium, and the environment. From a comprehensive perspective, the friction coefficient and the changes in its value are most affected by the weather conditions of the road surface, that is, rainfall, snow, ice, and others. These conditions make the road surface slippery and lead to abrupt decreases in the friction coefficient [1]. RSC sensing is used to detect changes in any of the factors listed above, realises the inverse of the friction coefficient to provide a reference for vehicular driving braking behaviour, and avoids potentially dangerous accidents.

The current autonomous driving technology is undergoing rapid developments in conjunction with the progressive development of perception technology and algorithms. Vehicular obstacle avoidance has a greater impact on driving safety and solutions to this problem is increasing. Based on this, the problem of RSC sensing is gradually being magnified and the research on this technology is slightly less, which is making it become a shortcoming in the development of autonomous driving which needs to be addressed effectively. At present, the mainstream sensors of autonomous driving are LIDAR (LIght Detection And Ranging), camera, millimeter-wave radar, ultrasonic radar, acceleration sensors, and others, which provide data support for obstacle avoidance, active safety, and speed control, but lack the function of RSC perception and cannot detect the road friction coefficient. The inability to sense RSC during road driving can affect the vehicular braking strategy, change the braking distance, and cause traffic accidents, such as crashes and side slides. Therefore, accurate assessment and detection of the road friction coefficient can provide a strong basis for vehicular decision-making, allow the conduct of research on RSC, and aid the improvement of travel safety.

Common RSC inspection methods include contact and non-contact measurements. About contact RSC inspection methods, the common international evaluation indices for road surface friction coefficient are the British pendulum number, side-way force coefficient, and braking force coefficient [2]. These three indicators are used to obtain the road parameters by measuring the coefficient of friction based on the use of traditional mechanical methods. In addition to the above methods, contact sensors are also available: capacitive, mechanical, fibre optic, flat film resonance, magnetostrictive, and piezoelectric resonance [3]. However, given the road vehicles, road surface materials, and weather, will (among others) affect the accuracy of the sensor. In this respect, contact sensors will have a short service life, high installation, and maintenance requirements, and will not be able to provide real-time measurements at the same time because the demand for RSC sensing in autonomous driving is mobile and portable. Accordingly, efficient, non-destructive, real-time measurements are needed to provide data support for driving and braking; contact measurements cannot meet the current demand for autonomous driving. Therefore, non-contact measurements are emerging gradually. Common non-contact RSC detection includes infrared spectroscopy, polarisation, radar detection, and computer vision. These four methods all use different principles to determine the conditions of the road surface. Non-contact RSC detection has become popular in recent years because of its advantages of portability and real-time, which meet the demand for real-time measurements of RSC in autonomous driving.

**Figure 1:**
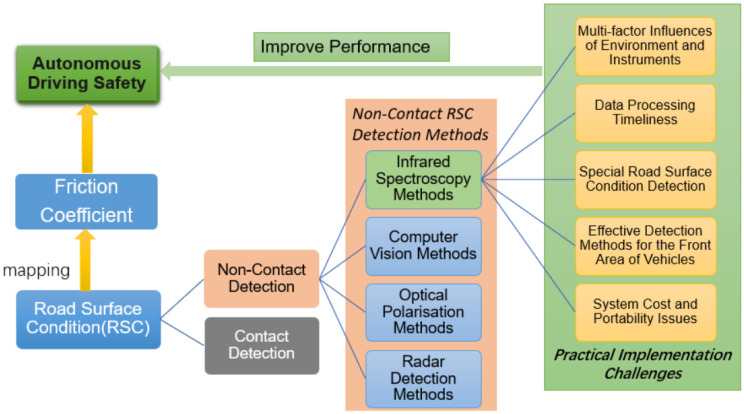
Structure diagram of this paper.

In this paper, based on the mapping relationship between the friction coefficient and the RSC; the factors affecting the RSC were introduced; the four non-contact detection methods and the current status of research were summarised and analysed; and proposals were raised to solve the problems that exist in the future practical applications. It is expected to provide reference for the research on RSC detection for the rapid development of autonomous driving. Section 2 introduces the influencing factors affecting road surface friction coefficient and the principles of mainstream, non-contact RSC detection methods; Section 3 systematically describes the current research and application status of non-contact detection; Section 4 compares the four RSC detection methods and suggests solutions and feasible research directions for the problems of infrared spectroscopy in the direction of practical road-condition detection. Section 5 summarises the existing RSC detection techniques and provides an outlook. The structure of the article is shown in Figure 1

## 2. Principles and Methods

**Figure 2:**
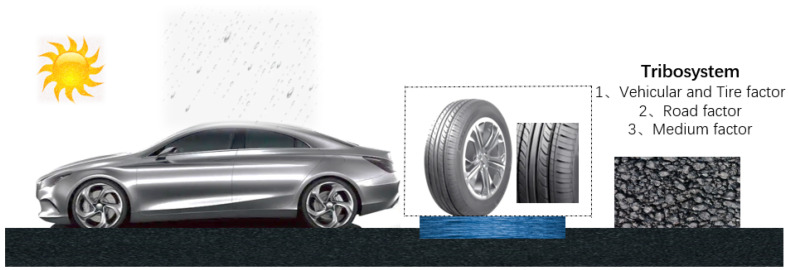
Tribosystem evaluated in this study.

The RSC affects the relative friction coefficient between the vehicle and the road surface during driving. Conducted research for vehicle–road surface friction is divided into two categories: experiment-based [4] and model-based. The conducted research based on the experimental method aims to establish the correlation between the data collected from multiple experiments and the friction coefficient; the research based on the model method aims to generalise the corresponding mathematical model, and the common ones are based on the vehicular dynamics model and the tire [5] as base models [6]. The factors affecting the friction coefficient between the tire and the road surface can be summarised according to the following three aspects: the tire, road surface, the system environment, and the road–tire medium [7,8], as shown in Figure 2. Among them, the influence of tires on the friction coefficient is mainly reflected in the tire type, tread, inflation pressure, and tire temperature. The current vehicular tire friction model is more mature, with high accuracy and repeatability. The influence of the road surface friction coefficient is mainly determined by the road material and texture of the grain surface [9]. Different road materials, such as asphalt and cement, have large differences in skid resistance, and road aging and cracks also have a large impact on road friction coefficient and skid resistance. Huang et al. [10] at the College of Transportation, Southeast University, studied the safe braking of unmanned vehicles based on the road surface friction characteristics and proposed a safe braking strategy for unmanned vehicles combined with the road surface texture characteristics. The unmanned vehicular model was established based on simulations, and vehicle braking characteristics, such as straight-line braking and turning braking emergency braking were studied. These provided a reference for the vehicular braking system and safety braking evaluation.

The influence of the system environment on the coefficient of friction is mainly influenced by temperature and season. The friction coefficient in dry RSC typically tends to have high values in winter and low values in the summer. The medium between the road surface and the tire mainly includes road contamination and rain, snow and ice, and other components that affect the friction coefficient of the road surface. When there is ice water and snow on the road, the contact between the tire and the road surface is prevented, the friction coefficient is reduced considerably, and the risk for accidents increases. Table 1 lists the approximate interval of the friction factor of the road surface in different RSC. Table 2 lists the effects of different road surface media on safe driving, and the combination of the two tables shows that different RSC have a significant effect on safe driving. Therefore, the study of RSC and the friction coefficient is necessary.

**Figure 3:**
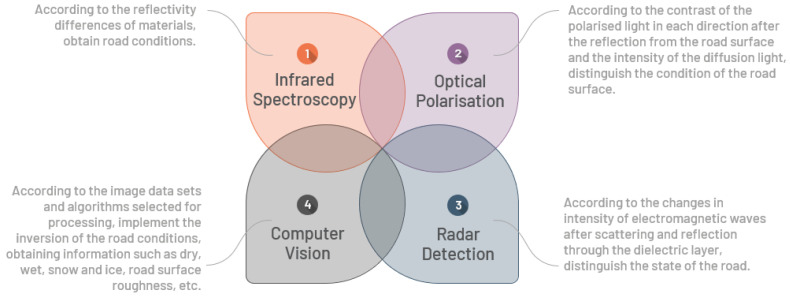
Principles of four non-contact RSC detection methods.

Non-contact inspection methods estimate the coefficient of friction by assessing the RSC to provide a robust parameter for vehicular driving. The RSC is typically defined based on different forms of road surface fluids in various conditions, i.e., dry, wet, icy, watery, and snowy. For non-contact road surface detection, the main detection methods include infrared spectroscopy, computer vision, optical polarisation, and radar methods. Figure 3 presents the principle of the four non-contact RSC detection methods.

## 3. Research and Application Status

This section presents a survey and overview of the current state of research and application of the four non-contact detection methods in Section 2.

### 3.1. Infrared Spectroscopy Methods

**Figure 4:**
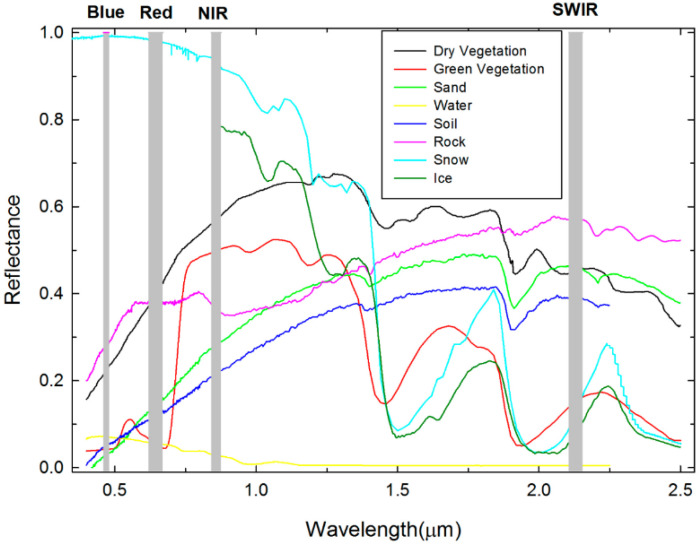
Spectral profiles obtained in the visible to near-infrared (NIR) bands of several typical land features (vegetation, sand, soil, water, and rock) extracted from the USGS spectral library [16].

The infrared spectrum is a fingerprint of the material’s composition. Water, ice, and snow have different reflectances to infrared light. The reflectance differences can be used as a basis to distinguish different road states. For RSC detection, we are most concerned about the road surface media, such as water, and its different physical state changes (water, ice, snow) in addition to the road material [14]. Owing to the large differences in absorption coefficients of different substances, the use of infrared spectroscopy for RSC detection typically selects several characteristic wavelengths that are sensitive to water, ice, and snow, and uses their specific absorption characteristics as the basis for discrimination [15]. Figure 4 shows the spectral profiles of several typical land features (vegetation, sand, soil, water, and rock) extracted from the USGS(United States Geological Survey) spectral library in the visible to near-infrared (NIR) bands.

**Figure 5:**
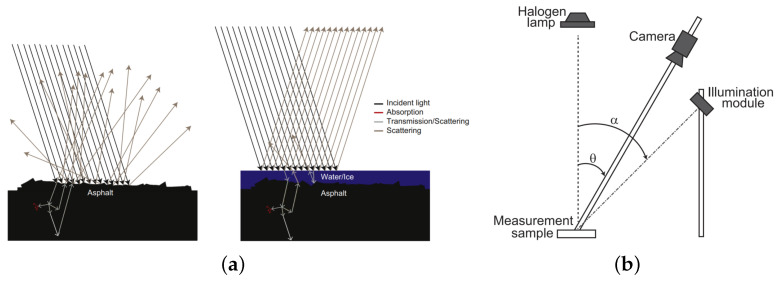
(**a**) Scattering of light incident on rough and smooth road surfaces. (**b**) Experimental setup with a halogen lamp (placed directly above the sample), a camera with an adjustable aperture lens, and an illumination module respectively mounted at angles equal to 30 and 45 degrees diagonally [17].

Casselgren [17] at the Luleå University of Technology(LTU), Sweden, used three characteristic wavelengths, namely, 980 nm, 1310 nm, and 1550 nm, with a FLIR SC7100 indium gallium arsenic (InGaAs) camera and a halogen lamp to simulate actual environmental applications with ambient light. A measurement scheme for handling ambient light was implemented that enabled RSC classification based on NIR images in real environments. The system can distinguish between dry, wet, damp, frosty, icy, and snowy road surfaces. The detection principle and experimental method are shown in Figure 5. Another study published by the same author [18] used two characteristic wavelengths, namely, 1310 nm and 1550 nm, to achieve data accuracies greater than 90% for water, snow, and icing conditions on the road, and for parameters, such as the thicknesses of water film, ice, and snow.

**Figure 6:**
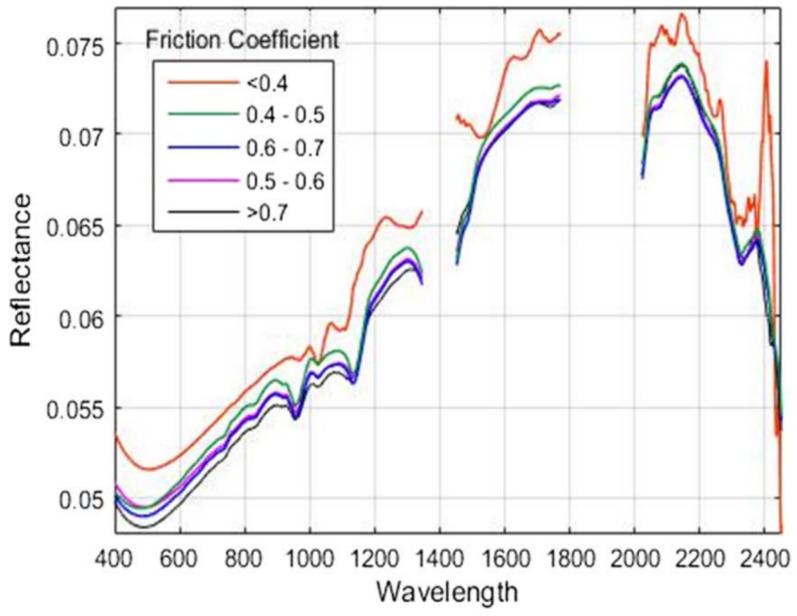
Asphalt spectra with ordered friction coefficient values reproduced from [19].

Carmon et al. [19] used a portable feature spectroradiometer (ASD) to identify road conditions by predicting the dynamic friction coefficient of asphalt in conjunction with the use of reflectance spectral data. The method used eight different spectral processing approaches to pre-process the data, which were: smoothing, MSC, SNV, absorbance, removal of continuous spectra, first-order derivative, second-order derivative, and final smoothing. The data from each pre-processing method were processed to create a data set. All data were modelled by using partial least squares and artificial neural networks (ANNs). After the data size was reduced with PCA, a system developed at the Remote Sensing Laboratory at the Tel Aviv University called ‘PARACUDA’ was used for fast automatic grouping and modelling. The final result achieved an accuracy equal to 0.845 between actual and predicted friction values (*p* < 0.0001). Figure 6 shows the average spectra of asphalt at different values of friction coefficients. Results show that the spectral characteristics of the asphalt road are influenced by the variation of the friction values.

Xu [20] at the Xi’an Institute of Optical Precision Machinery(XIOPM), Chinese Academy of Sciences(CAS), selected two wavelength bands at 1064 nm and 1550 nm, and used dual-wavelength coupling measurements to solve several difficulties associated with the sensor. This process successfully produced six sets of road-weather detection equipment that can realise all-weather monitoring of dry, wet, icy, and snowy RSC. Accordingly, the expected design requirements after experimental tests were reached; these can meet the existing highway requirements. Wang [21] at Zhejiang University(ZJU) chose the dual-light-source method based on infrared scattering spectra at two wavelengths, namely, 880 nm and 950 nm, to detect the RSC; derived and analysed the entire optical system, and designed the circuit structure of the entire system as well as the mechanical structure to combine it with the optical system. Based on the different absorption coefficients of water, ice, and snow to three specific wavelengths of light, Hao [22] and others from the Chinese Academy of Testing Technology developed a non-contact, remote sensing, RSC detection, and early-warning system independently by using infrared remote sensing and multispectral measurement techniques. The results were verified by several field tests to be accurate and reliable, with considerable safety and economic benefits, and have been successfully put into use on several highways in China with an accuracy rate of 100%.

**Figure 7:**
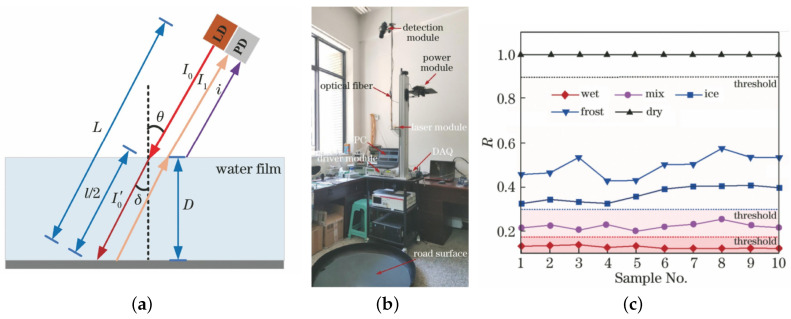
System used for water film thickness detection studied by Wu. (**a**) Mathematical model, (**b**) experimental set-up, and (**c**) relative reflectance measurements of each state when λ = 1550 nm, L = 3 m, and θ = $20^{\circ }$ [23].

Based on the characteristics of infrared reflectance spectra of water and ice, Wu [23] at the Chongqing Key Laboratory of Fiber Optic Sensing and Photoelectric Detection at Chongqing University of Technology(CQUT) selected the 1310 nm and 1550 nm bands to study a mathematical model and detection method for water film thickness detection and ice and water state detection discrimination and used Lambert’s law to process the reflected light intensity measured by their system to obtain the water film thickness. To calibrate accurately the water film thickness, two simulated polyester fibre road with similar infrared reflectances to those of asphalt road surface were used as the measured targets, and the actual water film thickness was obtained by measuring the water injection volume. Based on this, a road surface weather-sensing system was established, the optical structure was optimised, and the relative reflectance $R=(I/I_{dry})$ was defined as the discriminant; correspondingly, the water film thickness measurement and the RSC discriminant threshold parameters were used to distinguish the RSC. The experimental results showed that the measured distance of the system can reach 3∼5 m, and the measurement error of the water film thickness was less than 0.1 mm at 9 mm. Figure 7 shows the measurement principle, experimental set-up, and some results.

Table 3 summarises the characteristic wavelengths of the studies listed above. In addition to near-infrared spectral sensing methods, long-wave infrared temperature measurement techniques were also applied in RSC detections. Infrared temperature detection sensors generally operate in the 8–14 μm band, and different RSC have different target temperatures and different radiation energies, thus resulting in differences in the infrared signals received by the detectors. Accordingly, the RSC are judged by analysing the temperature signal changes [15]. However, infrared sensing temperature measurement technology is susceptible to ambient temperature, airflow, dust, and other factors, while the emissivity of the object and the temperature measurement distance both affect the measured results. Other parameters, such as humidity and wind speed, were also added to form a road-weather station, and thus differentiate the road surface.

In addition to the detection with the use of spectral instruments at the sites mentioned above, long-range remote sensing detection methods can also be used [24,25], which are characterised by large area coverage to obtain the macroscopic RSC distribution, but the resolution of the data is low. Jin et al. [26] at the Institute of Remote Sensing and GIS, Peking University, studied the remote sensing monitoring schemes of road deterioration by using the spectral characteristics of road surfaces. The surface reflection data of the road were obtained by using an ASD portable feature spectral radiometer. By analysing the spectral reflection and absorption characteristics of different spectral bands, the spectral response change pattern of an asphalt road during the aging process was explored. The spectrum of the new asphalt showed an increasing trend in the range of 400–2100 nm; as the severity of the aging effects of the asphalt road surface became more prominent, the rate of increase of the trend decreased. The old road surface exhibited a large increase in the range of 400–900 nm, and the absorption characteristics at 2300 nm became less obvious. Meanwhile, a spectral index model reflecting the health of asphalt roads was established based on this. This model selected the 450–510 nm and 770–895 nm bands as the parameters of the asphalt aging index and constructed four spectral index models, namely, the difference health index (DHI), ratio health index, normalised DHI, and logarithmic health index to reflect the aging condition of the road. Evaluation of these models led to the conclusion that the logarithmic health index had the best effect, and can achieve the detection of road health conditions by using high-spatial-resolution and satellite multi-spectral data.

**Figure 8:**
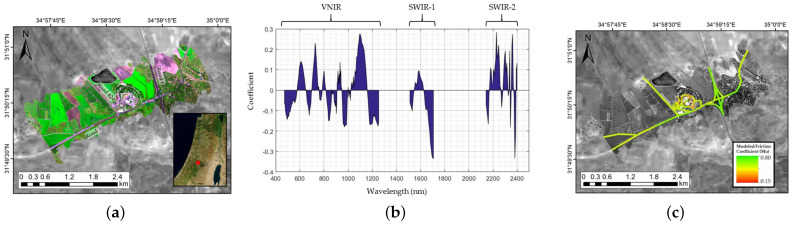
(**a**) Location selection, (**b**) coefficient spectra of the band weights in the model, and (**c**) predicted friction coefficient values for the study site map [27].

Carmon et al. [27] projected SPECIM AisaFenix 1K hyperspectral pixel data to the same background gray-scale image from sentinel-2. Friction coefficients of road surface conditions were modelled by the information on road reflectance coefficients which was extracted from the images. The spectral radiance data were processed using a ground calibration target and an atmospheric correction package to obtain road surface reflectance to form its final dataset of field friction measurement points with asphalt pixels and geographic corrections. A partial least squares regression model was developed. Spectral assignment analysis was used to identify the spectral bands with the highest weights in the model and their possible assignments were explored. Finally, the obtained model was applied to the hyperspectral images to invert the friction values for each road image element. The study demonstrates the feasibility of using aerial hyperspectral imagery with ground friction coefficient measurements to provide a broader overview of road conditions. Figure 8 shows a friction map of the road prediction study site obtained by Carmon through the direct application of the developed model to a hyperspectral data cube, which creates a continuous projection of the friction values of the road grid throughout the scene.

**Figure 9:**
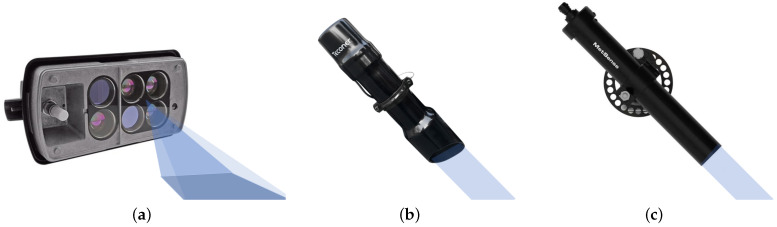
(**a**) MARWIS-UMB RSC sensor from LUFFT (Germany) [28], (**b**) RCM411™ intelligent optical RSC patrol system from Teconer (Finland) [29], and (**c**) MetRoad from MetSense (Sweden) [30].

Vehicle-mounted sensors based on infrared spectroscopy detection schemes can be either suspended on each side of the body or the roof of the vehicle and can obtain road parameters in real time while the vehicle is moving. The current market for vehicle-mounted road sensors is dominated by a few European products, as shown in Table 4. The most common RSC detection product on the market is the MARWIS-UMB (Mobile Advanced Road Weather Information Sensor) and RCM411 (Runway Condition Monitor) vehicle-mounted RSC sensor. Figure 9 shows products that use infrared spectroscopy to detect RSC. The principle is based on the determination of RSC through the absorption of water, ice, and snow at various levels in certain wave bands, which can be integrated into existing traffic networks.

To compare the performance of MARWIS and RCM411, Brustad et al. [31] at the University of Tromso, Norway, conducted field experiments and completed long-distance laboratory and road tests. The test conditions included weather to heavy snow, and the road surface conditions included ice, snow, damp, wet, mud, and dry conditions. Test variables included the friction coefficient, water film thickness, and road gauge temperature. The test results showed that RCM411/MARWIS for the three variables were consistent. RCM411 was more stable, and its value was close to the predicted values. Although MARWIS had some incorrect readings, MARWIS was more sensitive to small changes in RSC, especially to the thickness of the water film.

**Figure 10:**
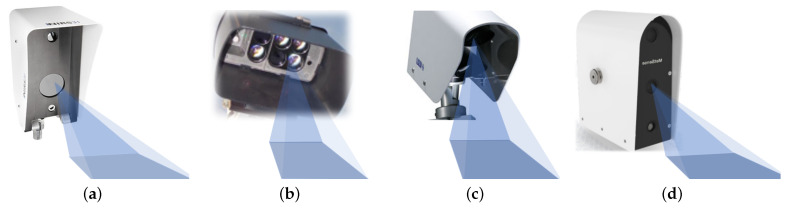
Typical, fixed products: (**a**) NIRS31-UMB remote sensing RSC sensor from LUFFT (Germany) [32], (**b**) StaRWIS-UMB remote sensing RSC sensor from LUFFT Germany) [33], (**c**) DSC111 road surface sensor from Vaisala (Finland) [34], and (**d**) 2DRoad from Metsense (Sweden) [35].

In addition to vehicular-mounted sensors, many stationary sensors use infrared spectroscopy; these are typically installed on road masts at specific angles and heights, as shown in Figure 10. In autonomous driving roads, vehicles can obtain road information detected by stationary sensors via the Internet of Vehicles to provide a reference for autonomous driving. Compared with in-vehicle products, the functions and configurations of stationary RSC sensors are better than in-vehicle products, but the prices are typically higher. At the same time, owing to the suspension height and field-of-view, the detection range is much higher than that of the vehicle-mounted detection sensor. Its measurement principle is the same as that of vehicle-mounted products.

For stationary road sensors, there are similar alternatives that are considerably lower in price than the typical products listed above, but with slightly worse performance and stability characteristics. For vehicle-mounted road sensors, research institutions and companies for RSC detection are mainly distributed in northern Europe, such as Finland and Germany, owing to climatic conditions and usage requirements. At present, the research in northern European countries is more advanced, but its core technology is more valuable and is coupled with agency and maintenance problems; correspondingly, there are fewer sensor users. At the same time, owing to the small number of product users, most areas do not have services, such as data support and sharing. At present, with the rapid development of autonomous driving technology, the demand for RSC perception is increasing rapidly, and related products and models with high performance and low prices are urgently needed to interpret the surrounding environment. In addition, most of the existing RSC detection methods experience real-time problems. There are few studies on the detection of friction coefficient at the mobile terminal of automobiles, and there is a large demand gap, especially in areas with complex RSC in areas at high altitudes and winters.

### 3.2. Computer Vision Methods

In recent years, machine and deep learning have developed tremendously. By training a large number of learning samples, the mapping of images and RSC can be achieved without the need for precise mathematical formulas. Convolutional neural networks (CNNs) are extensively used in vision-based RSC recognition algorithms because of their powerful capabilities and high robustness to scaling and tilting of geometric target shapes. In addition, there are many other network structures, such as support vector machines (SVMs), artificial neural network(ANN), deep convolutional neural networks(DCNN), recurrent neural network(RNN), long short term memory(LSTM), LSTM-RNNs [36], and others, which have been applied to RSC classification.

Common computer vision methods for the perception of RSC are generally used to estimate the friction coefficient of the road surface by determining the state of water, ice and snow on the road surface.

**Figure 11:**
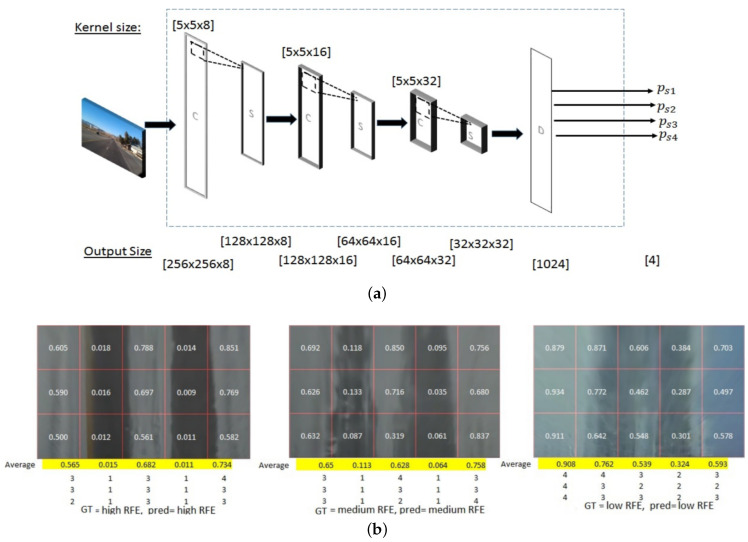
(**a**) Example of convolutional neural network architecture with three continuous convolution/sub-sampling C-S layers (CS3) followed by four output class dense D flattening layers. (**b**) Example of road chunking quantisation and road reflectance classification results [37].

Roychowdhury et al. [37] estimation of real-time road friction coefficients by using machine learning. They proposed a two-stage approach for estimating RSC by indirect reflectance based on the use of forward-looking camera images captured from vehicles. In phase 1, a convolutional neural network model architecture was implemented to learn region-specific features for RSC classification. The discovery of texture-based features from the driving surface, the sky, and the surrounding environment were determined to be independent regions for RSC classification, such as dry, wet, snow, mud, and ice. In phase 2, the driveable surface of the lane was segmented into [5 × 3] blocks by its established model, and the segmented blocks were classified and quantified to separate the image reflectance with high, medium, and low reflectance. Part of the results are shown in Figure 11b below. The proposed method achieves an average accuracy of 97% in the first and second stages of RSC classifications.

Vosahlik et al. [38], implemented a visual predictor of the friction coefficient of the road surface in front of the vehicle. An image recognition neural network was used for training by using an SVM method that replaced the tedious, error-prone, and subjective manual annotations. The visual predictor was trained by using approximately 3000 images and was validated on 800 test images. The training images were automatically labelled by surface friction estimates of the vehicular response during normal driving. The frictional parameters of the tire-road interface were estimated by using the unbounded Kalman filter algorithm. Finally, the entire process was validated by using an experimental sub-scale platform based on a real-world driving scenario. The experimental results achieved a correlation of 0.98 between the visual predictions and the surface friction estimates.

Tian et al. [39] proposed a data-level fusion recognition framework that combined semantic segmentation, shadow detection, and a classification model to classify road conditions as dry/wet asphalt and snow/ice. By using the fusion approach, the accuracy exceeded 92% on the test set without shaded scenes. To verify that the classification results exhibit a large improvement as a function of the braking distance, the results of its classification and fusion were simulated on a specific data set with the anti-lock braking system model. The results showed that the vehicle was able to reduce the braking distance by 25.8% and 22.9% in snow and dry-to-snow conditions, respectively. This has significant implications for improving the performance of active safety systems.

Nolte et al. [40] used ResNet50 for RSC classification, and obtained a 92% accuracy. Busch et al. [41] implemented the use of neural networks to classify road surface and weather-related conditions and evaluated the performance of three networks, namely, Inception-v3, GoogLeNet, and the smaller SqueezeNet under DCNN, based on the F1 score. SqueezeNet architecture had the highest F1 score of 95.36%, where the F1 score was defined as Equation (1).

(1) $$F_{1}=2\times \frac{precision\times recall}{precision+recall},precision=\frac{TP}{TP+FP},recall=\frac{TP}{TP+FN}$$

Shimano et al. [42] derived and analysed a spectral model of a wet-road surface by using the principle according to which road moisture causes image colour changes; recovery of the original surface colour and wetness from a single observation were achieved, thus verifying the effectiveness of the method for estimating road friction coefficients from road surface moisture and colour.

**Figure 12:**
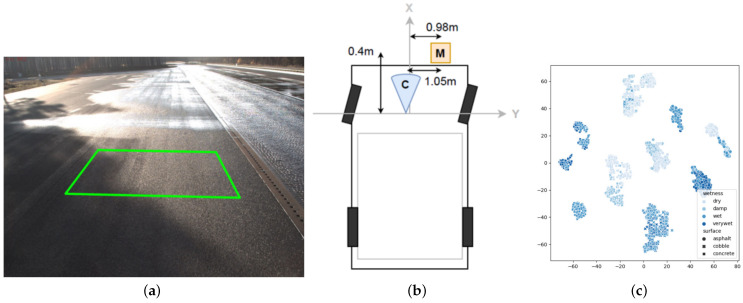
Kai Cordes dataset collection process. (**a**) Schematic representation of the area from which the data set was collected. (**b**) Location of the detection equipment mounted on the vehicle. The orange square Marwis is located at the bottom right of the vehicle, and the blue cone C shows the camera mounted at a fixed height above the car; (**c**) Visualisation and analysis of the RoadSaW dataset based on the use of t-SNE. The three surfaces, namely, asphalt, cobble, and concrete, are indicated by different markers. The four subcategories, namely, dry, wet, wet, and very wet, are shown with four shades of blue [43].

Current CV methods are used to classify RSC and are often trained on a large number of images to classify road dryness, rain, ice, and snow, and to predict road friction. This method currently has a partial data set, as shown in Table 5 below. In 2022, Cordes et al. [43] made public their newly built dataset, which is based on cameras and real road data measured by MARWIS to estimate the friction coefficients. Image video and MARWIS measurements were provided for different detection distances and area sizes. A basis for future road friction coefficient estimation was provided. Figure 12 shows a selection of the detection roads from their acquisition data set, the experimental apparatus installation angle, and some of the data validation results.

There are also many researchers who have processed image information to obtain texture information and roughness of asphalt road surfaces to obtain an estimate of the friction coefficient of the road surface. For example, Ivana Pranji et al. [45] summarised a study on the establishment of friction coefficients with road surfaces based on texture features, and Ozcan et al. [46] evaluated the quality and accuracy of a road surface roughness measurement system based on the use of a camera. Amthor et al. [47] proposed a method for classifying RSC through the use of a monocular camera based on a reflection model by fitting the input image as a reflection map, and by combining the texture features of the original map, with good real-time results. Tumen et al. [44] designed a new CNN model to determine road type and road quality; accuracies equal to 91.41% for road type identification, and 91.07% for pothole road-smooth road differentiation were achieved.

Most autonomous vehicles are equipped with cameras for visual navigation and obstacle detection. The use of data collected by front-view cameras for the prediction of the friction coefficient of the road ahead is typically based on image data from existing sensors, which is advantageous in terms of cost control. However, the computer vision method is strongly dependent on the data set, the quality of the data set has a strong influence on the inversion of the friction coefficient, and the method requires high-processor performance [48]. Conversely, light sources are a prominent issue in the computer vision method case, and the absence of illumination at night and the interference of various lighting conditions with ambient light can have an impact on the results. The aforementioned issues constrain the application of computer vision methods in the detection of road surface conditions. With the rapid development of machine and deep learning, a road surface detection method based on image video and various advanced classification algorithms intended to fuse meteorological data and vehicle models will potentially overcome the road-detection problems associated with computer vision methods. This constitutes one of the popular directions of current research.

### 3.3. Optical Polarisation Methods

Light waves are a type of electromagnetic wave, and polarisation is one of their important characteristics. As water and ice molecules are not strictly aligned and are anisotropic, the incident beam of light will break up into two types of polarised light with different propagation speeds, and the intensity of the detected polarisation will change. When light is directed at a particular angle (angle of divergence or Brewster’s angle), the reflected light becomes perpendicular to the refracted light and can be used to determine whether there is water or ice on the road surface. The polarisation method uses a photodetector to measure the intensity of the reflected and scattered light and uses the horizontal and vertical components of the reflected light to obtain the polarisation parameters that can be used to distinguish between dry and waterlogged conditions, and between wet and ice based on the intensity of the scattered light. The polarisation method does not require a light source and can reduce costs. However, the results can only be measured qualitatively, and a qualitative description of road adherence needs to be combined with other measurement methods. The difference between the refractive indices of water and ice is small, and the difference in angle of departure is also small; thus, the accurate differentiation between water and ice requires a high degree of precision in terms of the installation angle of the detection instrument.

**Figure 13:**
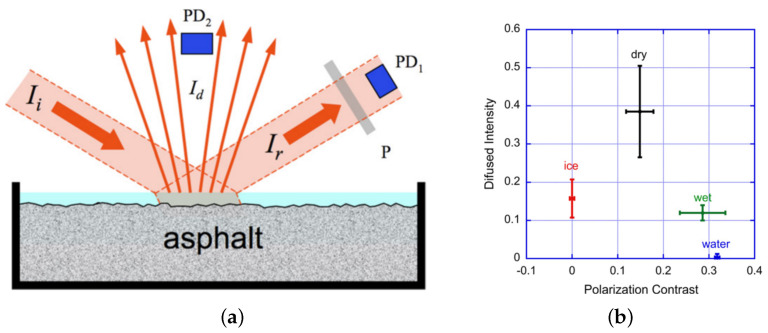
(**a**) Schematic of the Colace polarisation experiment. (**b**) Comparison of diffuse reflectance intensity and polarisation [49].

Colace et al. [49] used an indium gallium arsenide (InGaAs) infrared detector to measure diffuse, reflected light subjected to near-infrared illumination, and extracted the contrast after polarised light reflection. Several tests were conducted on various types and thicknesses of asphalt. The conclusions showed that the system could identify dry, water-layered, wet, and asphalt with ice, and demonstrated that the system was robust. The setup is shown in Figure 13a. PD1 and PD2 are InGaAs photodiodes, and P is the analyser. Two InGaAs photodiodes measure the intensity of the two polarisation components of reflected light $I_{r}$ (via PD1 and the analyser) and diffuse light $I_{d}$ (via PD2) respectively. The resulting surface polarisation contrast constitutes reliable data for identifying normal, ice, and wet/water conditions, but it needs data from diffuse light to distinguish between wet and water-covered roads. Figure 13b below shows some of its experimental results, which are the diffuse reflected intensity and polarisation contrast.

Yuan [50] at Huazhong University of Science and Technology proposed a method that uses near-infrared tungsten halogen lamps for illumination and rotates the polariser to collect polarisation intensity images in four directions. The linear polarisation degree is calculated from these images to identify the state of the RSC. Results showed that the line polarisation is larger when the surface is icy, smaller when it is wet, and the smallest when it is dry. Results show that the line polarisation is larger when the surface is icy, smaller when it is wet, and the smallest when it is dry. The accuracy associated with the identification of the RSC was achieved 90%. However, this method requires artificial rotation of the polariser each time, and it is difficult to ensure the same angle. Shuya [51] developed a road surface icing detection system, set the wave band at 780–1150 nm, assessed the road icing status by detecting the linear polarisation of the reflected light, judged the ice thickness by detecting the size of the reflected laser spot, and built a test system to verify the feasibility of the principle.

**Figure 14:**
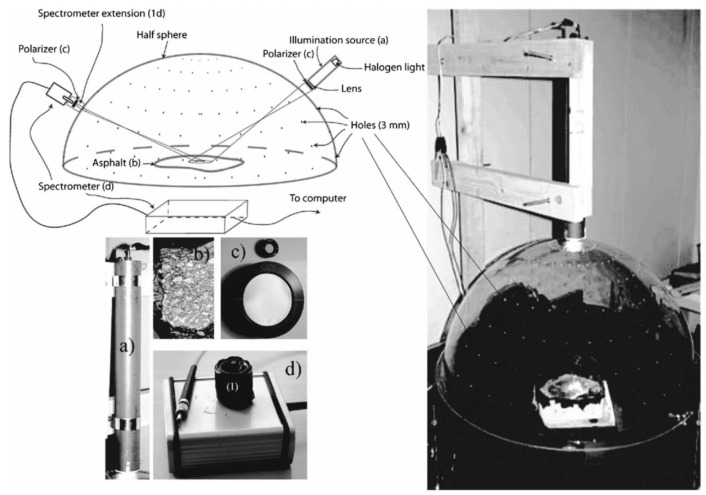
Diagram of the experimental setup of Casselgren. **Left top** is a sketch over the measuring procedure. Right is the experimental setup. A hemispherical Plexiglass hood was used. Holes were punched at 10-degree intervals in the longitude and latitude directions of the hood with a diameter of 3 mm, and the hood was placed over the road asphalt sample (**left bottom** (**b**)). A polariser (**left bottom** (**c**)) was placed in front of the light source (**left bottom** (**a**)). The spectrometer with the extension (**left bottom** (**d**)), and the direction of polarisation was adjusted both horizontally and vertically [53].

Finnish researcher Jokela [52] distinguished between RSC by combining optical polarisation and image grain size analysis, and demonstrated accuracies greater than 90% for ice analysis. Casselgren [53] at Luleå University of Technology, Sweden, proposed a new polarisation method by using the anisotropy of ice bi-refringent crystals to effectively differentiate between nine states of the road surface, such as dry, wet, and snowy, as shown in Figure 14. This device requires the rotation of the light source and detector at the same point, and it is thus not suitable for mounting on the shoulder of the road as it requires human intervention and is cumbersome.

Owing to the limitations of the polarisation method itself in terms of the high precision required of the instrument and the installation angle, most of the current research related to polarisation-related products and instruments is at the experimental principle prototype stage and has not been put into use on a large scale.

### 3.4. Radar Detection Methods

In RSC detection, radars are mainly classified into three types, including millimeter-wave radar, LIDAR, and ultrasonic radar [54]. The radars used for road surface detection are the millimeter-wave radar and LIDAR. Millimetre-wave radar is mainly based on the intensity changes of electromagnetic waves after scattering and reflection through the water layer, ice, and snow of the road surface to distinguish the state of the road surface. LIDAR is used to construct a three-dimensional point cloud of the road surface by detecting the three-dimensional morphology of the road surface [55]; it can detect the roughness of the road surface, and thus evaluates the friction coefficient of the road surface. Ultrasonic radar works based on the change of acoustic parameters after ultrasonic waves pass through different road states to identify internal road defects, cracks, and other conditions. The three radar methods have the ability of RSC detection, among which the millimeter-wave radar detection method can detect small angles, but the detection distance performance is not as good as LIDAR, the cost of LIDAR to measure road surface roughness is too high, and ultrasonic radar cannot achieve accurate detection of road weather conditions. Meanwhile, the accuracies of the three radar detection data sets are limited, and there are only a few studies that use radar detection methods to achieve RSC detection.

Asuzu et al. [56] presented the method of radar backscatter measurements in various RSC. Backscatter measurements of asphalt road surfaces were obtained for stationary and moving radar platforms in dry, rainy, snowy, and muddy conditions with the help of millimeter-wave automotive radar. The effect of RSC on radar backscatter was examined, and the feasibility of the RSC identification scheme was demonstrated.

**Figure 15:**
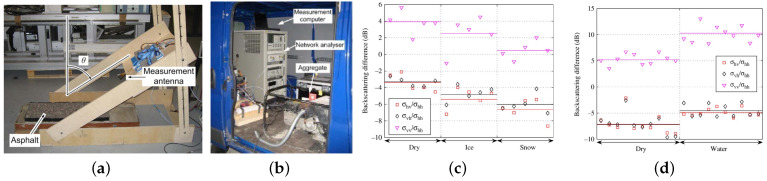
(**a**) Detection device. Horn antenna mounted on the rotating arm points to the asphalt placed on the floor and is used for detection. (**b**) Placement of the detection device on the truck. (**c**) Markers indicate individual measurements, and solid lines indicate the average of the corresponding RSC and polarisation values showing the backscatter ratios for different polarisations; the graphs show the backscatter versus polarisation normalisation in different polarisations for dry, ice, and snow asphalt road. (**d**) Backscatter versus polarisation normalised backscatter for dry and wet asphalt at different polarisations [57].

Viikari [57] investigated 24 GHz automotive radar technologies by using millimeter-wave radar and studied the backscattering characteristics of asphalt in different conditions with the detection setup shown in Figure 15. Millimeter-wave radar has a better ability to penetrate dust and smoke compared with LIDAR, and it can work in extreme weather conditions. This study investigated the effect of water on the backscattering characteristics of asphalt by using a surface scattering model to detect low-friction points by comparing the backscattering signals in different polarisations. The results showed that it is feasible to use radar to detect RSC.

Darapu et al. [58] proposed a road recognition technique based on frequency modulated continuous wave remote vehicle radar operated at 77 GHz. The cepstral coefficients calculated from the backscattered signals were analysed by using classifiers, such as decision trees and SVM. Field tests were conducted at Kondapur (Telangana), and the system achieved a prediction percentage which was more than 90%.

A review paper by Bystrov [59] describes different frequency radars in the frequency range of 10–79 GHz applied to road surface classifications in the car driving cases. His research analysis suggests that polarised radar is an effective way to classify RSC. The data obtained at different frequencies and angles of incidence can be combined to improve the accuracy of the detection system. However, owing to the complexity of the RSC and the difficulties associated with the interpretation of the obtained information, the present research on the classification of RSC based on the use of radar is limited to the theory of signal backscattering from various surfaces and its experimental validation in many experiments.

Radar is extensively used for obstacle detection in autonomous driving. Radar has a limited ability to recognise road-surface media; thus, it is rarely used to detect road surface conditions. The detection distances of different radars vary extensively, and their application scenarios also vary. The vulnerability of these radars to adverse weather conditions, such as light and rain, snow, and fog, and their high costs also affect large-scale applications. The future fusion of radar data with images and spectral information will make up for the lack of sensors themselves and will provide abundant information for autonomous driving.

## 4. Practical Implementation Challenges of the Infrared Spectroscopy Method for Road-Surface Condition Detection

From the current field of autonomous driving, the relevance of RSC to the issue of driving safety has become increasingly prominent with the enhancement of road-detection capabilities. The impact of RSC on vehicular driving is mainly reflected in the changes in road friction coefficient, which in turn reflects the degree of skid resistance of the road surface. Therefore, the need for RSC sensing has become increasingly urgent. To obtain the RSC information (i.e., the road friction coefficient), this study analyses the advantages and disadvantages of the above four detection methods, as listed in Table 6.

Infrared spectroscopy-based road surface condition detection can add a new dimension of data for autonomous vehicles and provide a new sensory ‘touch’ to autonomous driving. Additionally, the road data measured by infrared spectroscopy can provide a friction coefficient reference for autonomous driving and strong data support for safe braking and decision-making. In addition, they also provide a reference to improve the driving experience and maintain the body smooth to a certain extent. Overall, infrared spectroscopy can sense the material composition, and the type of road medium and liquid thickness has the advantages of real-time and high sensitivity and possesses great potential and broad development prospects. The following is an analysis of some technical challenges and problems in the future practical application and large-scale popularisation of the method of spectroscopy. This analysis provides reference solutions for the capacity enhancement and application popularisation of RSC detection.

### 4.1. Multi-Factor Influences of Environment and Instruments

RSC detection is mainly affected by two factors: environment and instrumentation. The environment has a greater impact on the detection of three aspects of light, temperature, and air medium.

For optical non-contact RSC detection instruments, the impact of ambient light on instrument detection is mainly reflected in the aspects of day and night, four seasons, ambient shadows, and strong specular reflections. The detection time affects the angle of incidence of sunlight, which changes the brightness of the pupil irradiation of the detection instrument, and the ambient shadow reduces the target reflectivity considerably, increases the redundant texture information of the target, and interferes with the perception ability and detection accuracy of the system. For the impact of ambient light on RSC detection, modulated active light source illumination and targeted de-improved data acquisition module approach is one of the ways to improve the above problems at the system level; conversely, algorithmic models can also be used to improve the robustness of the detection model and improve the sensing performance of the instrument.

The interference of temperature on RSC is reflected by the fact that temperature affects the skid resistance of the road and changes the correspondence between the medium, such as the water film and the friction coefficient [60]. Therefore, the measurement of temperature parameters and the establishment of temperature-based friction coefficient models are necessary for systems with high demand for road surface friction coefficient detection.

Air media, such as haze and rainy weather mainly affect the light propagation path and change the transmission medium transmission rate. By contrast, in the above special weather, slippery road surfaces and reduced visibility will increase considerably the incidence of road accidents. Therefore, the need for RSC sensing is urgent in the construction of road sensing instruments; thus, it is necessary to conduct targeted model research for the above special weather.

The influence of the instrument on the detection is mainly due to the detection angle, the detection distance, the light source, and the temperature of the instrument. The angle of detection and the sun or the angle of the light source affect the road surface reflectance parameters obtained by the instrument, which in turn affect the correspondence of the model. At the same time, light intensity varies as a squared multiple with distance, and for mobile road detection instruments, it is important to keep the detection distance as consistent as possible while moving. Conversely, a robust distance perception model needs to be established to accommodate the effects of terrain while the vehicle is moving [61]. For RSC detection instruments with active light sources, the stability of the light source is one of the key factors for accurate inversion of road parameters. In the process of instrument construction, full consideration needs to be given to the attenuation of the light source over an extended period by increasing the calibration function during the use of the instrument and enhancing the operability of the calibration function. To study the influence of temperature on the instrument, it is necessary to analyse the mechanism of temperature influence when constructing the instrument, establish a temperature calibration model, increase the testing and optimisation of the instrument, and further improve the accuracy of the detection data.

Exploring multi-factor interference solutions between the environment and the instrument, adding anti-jamming features to the instrument build to improve instrument robustness, and conducting targeted analysis of the data collected during the use of the instrument, with which the overall interference solution can be improved further in conjunction with the above analysis, can lead to considerable improvements in the accuracy and robustness of road detection instrument parameter measurements.

### 4.2. Data Processing Timeliness

The information on RSC in autonomous driving may come from multiple sensors, including cameras, radar, a global positioning system (GPS), and so on, and the system has to process these data to make judgments and decisions about the car’s driving performance. In this process, if the data processing speed is too slow, the system will not be able to make timely and effective judgments, especially in highway and complex RSC; additionally, the body will not make timely judgments. This will cause a large number of accidents. Therefore, the issue of data processing timeliness is very important. The current difficulty is the limited power consumption and arithmetic power of current in-vehicle devices, which cannot meet the demand for rapid detection. In the future, the following four points can be considered to solve this problem: (1) improve the arithmetic power of the vehicle; with the current hardware equipment and chip upgrades, the arithmetic power of the vehicle’s end will also gradually improve; (2) optimise the algorithm, adapt a more reasonable and efficient algorithm to solve the timeliness problem; (3) perform a data dimensionality reduction, select the main components of multi-source data for calculation, and optimise the recurring information and parameters; (4) consider combining vehicular network information to provide a priori information for RSC inversion and improve the algorithm’s operation speed.

Another frequent problem regarding timeliness is whether the data frame rate matches the needs of mobile detection. When measurements are conducted in motion, images, detectors, light sources, and so on need to be synchronised, and if a fixed frame rate detection is applied, the data acquisition interval will vary at different moving speeds. Therefore, it is necessary to adjust the detection frame rate according to the vehicle speed to avoid data redundancy at high-detection frame rates at low speeds, and to avoid information omission during high speeds to enhance detection efficiency. The detection frame rate adjustment strategy will be established after experimental analysis based on the sampling interval requirements, processor capability, and other factors.

### 4.3. Special Road Surface Condition Detection

In actual life, RSCs are many and vary, and there is a large number of interfering factors. At the same time, many extreme weather conditions affect considerably the RSC. The measurements of these RSC are a difficult problem at the moment as they include (1) the presence of black ice on the road surface that cannot be identified by the naked eye which will reduce considerably the friction performance of the road; (2) the presence of turbid muddy water on the road surface that leads to the inability to predict effectively the depth of water; (3) ice and snow road surface covered with ice or snow layer that prevent accurate determination of the thicknesses of the surface covers of these roads. The two main problems are the identification of the vehicular road media, and the detection of their depths and their effects on the friction coefficient of the surface. To address these two problems, firstly, the spectral method can be considered to detect the road surface components, collect a large number of different conditions of the road data, and establish a perfect inversion algorithm of the RSC. Based on these data, material classification and assessments can be achieved more accurately. Secondly, research can be conducted on the measurement of the thickness of media, such as the use of distance detection methods to measure water film or ice thicknesses, the use of optimised models to account for the effect of mud and water turbidity on the inversion of water film thickness, and the establishment of experimental correspondence between the friction coefficients of different water film/road ice thicknesses. In the future, these findings can be considered for applications related to the detection of actual RSC to inverse the friction coefficient of the road surface more accurately, and thus assist automated driving decisions.

### 4.4. Effective Detection Methods for the Front Area of Vehicles

The detection of RSC in front of the car, while it is in motion, is a necessary factor in making decisions. Two main issues influence the driving decision in front of the car area, namely, the distance and accuracy of the detection. This is similar to the effect of instrumentation in a multi-factor interference problem.

The main problems of the front-area detection include (1) long-distance detection problems at increasing detection distances, whereby the returned signal will be attenuated by the inverse square of the distance; (2) cameras and optical and other measuring instruments whose detection light source capacities are low, cannot cover long distances, and light will become more diffuse as a function of distance; (3) detection in the blind areas within the field of view and other factors; these problems will have an impact on the accuracy of the collected data, thus affecting the road, the accuracy of the condition detection, and the rationality of the decision.

The problems listed above can be solved in the following four ways. (1) By adjusting the light source, the problem associated with the weak detected source light intensity at long distances can be solved by increasing the intensity of the light source, or by the effective convergence of the light source. (2) With the use of front cameras and LIDAR, the rapid development of computer vision has provided an impetus to the detection of road conditions at long distances; additionally, LIDAR has advantages in long-distance detection. By combining multiple sensor data fusions, De Jong Yeong’s research [62] showed that sensor fusion helped overcome the shortcomings of individual sensors and helped build a continuous and stable model to accurately perceive the surrounding environment in various conditions, as shown in Table 7. (3) Consider interactive interconnection through vehicles, whereby the rear vehicle uses the driving pattern of the front vehicle to make predictions based on vehicular linkage. (4) Classify and conduct algorithmic optimisation based on a large amount of analysed data by using reasonable classification algorithms to distinguish road conditions, such as YOLO [63], SSD [64], PointNet [65], and others.

In the table, the ‘√’ symbol indicates that the sensor works as desired at the given factor; the ‘∼’ symbol indicates that the sensor performs fairly well at the given factor; the ‘×’ symbol indicates that the sensor does not operate well concerning the sensor’s factor. This table compares several key characteristics of the sensors, including detection range, resolution, detection distance accuracy, lighting conditions, and weather conditions. We can conclude that individual sensors work well only in specific areas, but in the face of complex driving environments, the result of multi-sensor fusion gives them all a substantial improvement in overall performance and a more accurate perception of the surrounding environment.

### 4.5. System Cost and Portability Issues

The current common mobile continuous road inspection equipment is more expensive and has a considerable impact on its large-scale popularity and use. The cost of the equipment is mainly attributed to precision instruments, such as optical accessories and cameras, as well as the property rights of the core technology. These detection instruments have high-hardware costs, and the specifications of the instruments in the autopilot favour aspects such as a smaller size, weight, and increased portability. The two proposed options include the optimisation of the hardware level of the RSC detection system, optimisation of the system layout, and reduction of the system cost. Consider the common RSC detectors MARWIS and RCM411 and other non-contact detection products as examples. Their prices are approximately equal to 50 times higher than those of the traditional instruments, such as the BM pendulum friction coefficient measuring instrument. Their principle of operation is based on the selection of the infrared wave band sensitive to the road surface, water, ice, and snow, based on the use of specific algorithms for the data of these wave bands to study and calculate the RSC. For application scenarios that do not require high-precision data, different solutions can be developed to achieve low-cost RSC detection. Specifically, for areas associated with a low probability to be affected by snow and ice, the road detection device will not be able to measure snow and ice, but will only classify the dry and wet road surface. Second, a combination of the use of the Internet-of-Things and the Internet-of-Vehicles, only the road’s and the car’s ends of the detection system of effective information uploaded to the server (via the integration of the vehicle’s detection parameters and other platform parameters) will achieve real-time detection of RSC, thus reducing the cost of a single sensor and computing needs.

## 5. Conclusions and Prospects

During the rapid development of autonomous driving in the current era, RSC detection has gradually become a shortcoming that restricts driving safety because of the lack of detection of road friction coefficient parameters, and the lack of information for decision-making in vehicular driving. With the increasing demand for autonomous driving for RSC information, the research of RSC detection is necessary and urgent, and the development of RSC information (obtained in real time and optimised for driving schemes and braking strategies) guarantees safe and comfortable autonomous driving. In this study, the recent research works on non-contact RSC detection were reviewed, the influencing factors of RSC were introduced, and four main principles and research statuses of non-contact RSC detection schemes were summarised, including infrared spectroscopy methods, computer vision methods, optical polarisation methods, and radar detection methods. Among them, infrared spectroscopy methods constitute effective means of non-contact RSC detection with their characteristics of rapid detection, high accuracy and precision, and practical possibility.

There are still technical limitations in the practical implementation of infrared spectroscopy for RSC detection. This paper presented a detailed analysis of the key technical issues of road surface detection based on infrared spectroscopy and proposed a reference solution. This study will help to improve the effectiveness of non-contact RSC detection and friction coefficient inversion. However, this study mainly focused on the analysis of non-contact RSC detection methods, which was based on the existing correspondence between RSC and friction coefficient, this correspondence was not described since there were only few studies. The research on this correspondence is of high research value, which will help increase the reliability of RSC detection and promote the driving safety.

In the future, the integration of multiple pieces of information to develop more accurate RSC detection models, solve the problems of multi-factor effects, improve the instrument detection timeliness, overcome the difficulties of special road detection cases, realise pre-vehicular RSC detection, achieve further reductions in system cost, and improve system portability, will help build a more comprehensive and accurate peripheral driving scene, thus improving driving safety and experience. In addition, the technology of RSC real-time sensing can also be extensively applied to a variety of scenarios, including exploration robotics, home/commercial robotics, road de-icing, and snow removal equipment, military road surveys. In the future, different systems can be developed for different application scenarios to meet their needs, thus acquiring high-economic value and research significance.

## Figures and Tables

**Table 1:** Friction coefficient at different RSC [11,12].

Vehicle–Road Medium-State	Dry	Damp	Wet	Black Ice	Snow	Thin Ice	Thick Ice
Friction coefficient	1∼0.8	∼0.75	∼0.65	∼0.50	∼0.40	∼0.35	∼0.2
Accident rate per million vehicles/km	1.6%	3.2%		8.0%		12.8%	

**Table 2:** Friction coefficients of road surface at different vehicular speeds [13].

Vehicle Speed	Dry Road	Wet Road	Mud	Ice
50 kmph	1	0.5	0.4	0.1
90 kmph	0.91	0.2	0.1	0.05
130 kmph	0.84	0.15	0.08	0.0

**Table 3:** Selection of spectra by personnel from various research institutions.

Research Institutions	Selected Spectrum (nm)
Jonsson, MIUN [15]	960, 1550, 1950
Casselgern, LTU [17]	980, 1310, 1550
Xu, XIOPM [20]	1064, 1550
Wang, ZJU [21]	880, 950
Wu, CQUT [23]	1310, 1550

**Table 4:** Comparison of typical vehicle-mounted RSC detection product parameters.

	LUFFT MARWIS-UMB [28]	Teconer RCM${}^{411}$ [29]	MetSense MetRoad Mobile [30]
Provide data	Road surface temperature, water film height, dew point temperature, RSC: dry, damp, wet, snow, ice; ice rate; friction coefficient, and road surface humidity.	RSC: dry, wet, damp, snow, ice, friction coefficient, water layer thickness, surface temperature.	RSC: dry, wet, damp, ice, slush, snow.
Size	110 mm × 200 mm × 100 mm	Length 270 mm, diameter 40 mm	298 mm × 50 mm × 50 mm
Weight	4 kg	0.7 kg	0.5 kg
Operating voltage	10–20 V	9–30 V	8–15 V
Operating temperature	−40–60 ${}^{\circ }$C	−30–50 ${}^{\circ }$C	−30–40 ${}^{\circ }$C
Accuracy of temperature measurements	±1.5${}^{\circ }$C	±0.3${}^{\circ }$C	±0.1${}^{\circ }$C
Temperature resolution	0.1${}^{\circ }$C	0.1${}^{\circ }$C	∼
Water film measurement accuracy (range)	0.1 μm, ( 0–6000 μm )	below 1 mm accuracy is 0.1 mm, above 1 mm accuracy is 10% detection thickness (0.03 mm–3 mm)	∼
dimensionless friction μ	0–1 resolution: 0.01	0–1	0–1
Sampling frequency	100 Hz	1 Hz	1 Hz
Installation height	1 m/2 m	∼	0.5 m

**Table 5:** Comparison of existing datasets in RSC detection [43].

Dataset	Image Source	Calibration	Annotation	Label Resolution	Wetnesses
Tumen et al. [44]	Google street view	×	Best guess	Full image	0
Roychowdhury et al. [37]	YouTube Video	×	Best guess	Full image	4
David Vosahlik et al. [38]	Test track recording	√	Established data	ROI, BEV	3/5
Busch et al. [40]	Compiled	×	Best guess	Full image	2
RoadSaW et al. [43]	Test track recording	√	MARWIS	ROI, BEV	2/4/∞

**Table 6:** Comparison of four RSC detection methods.

	Advantages	Disadvantages
Infrared spectroscopy method	High accuracy and fast recognition; strong recognition of special road conditions, such as snow and ice; related products are already available.	The light source and background have a large impact on it with high-technical requirements and high product prices.
Computer vision method	A wide selection of cameras model, hardware devices with relatively mature technologies, low prices, and high possibility of re-use.	Accuracy and timeliness are strongly influenced by the classifier and training scheme; images are susceptible to lighting conditions and camera resolution limitations; high-arithmetic power requirement.
The optical polarisation method	The principle is relatively simple and easy to implement.	Most of the product instrumentation research is at the principal prototype stage; increased requirements for installation angle and mechanical structure; the environmental changes have a substantial impact on the detected results; practicality needs further evaluation and limitations exist.
Radar detection method	There are many types of radar, and the technology is more mature; obstacles can be identified effectively; high possibility of reuse.	Special RSC, such as snow and ice are weakly identified, and there are fewer studies and data at this stage based on the use of radar to classify RSC due to the complexity of the RSC and the difficulty of interpreting the information obtained from them.

**Table 7:** Technical characteristics of sensors in autonomous driving and their performances subject to the influences of external factors [62].

Factors	Camera	LIDAR	Spectroscopy	Fusion
Range	∼	∼	∼	√
Resolution	√	∼	√	√
Distance accuracy	∼	√	∼	√
Illumination conditions	×	√	×	√
Weather conditions	×	∼	√	√

## Data Availability

Not applicable.

## Abbreviations

The following abbreviations are used in this manuscript:

RSC	Road surface condition
LIDAR	Light detection and ranging
BPN	British pendulum number
SFC	Side-way force coefficient
BFC	Braking force coefficient
NIR	Near infrared
USGS	United States Geological Survey
LSTM	Long short term memory
CAS	Chinese Academy of Sciences
XIOPM	Xi’an Institute of Optics and Precision Mechanics
ZJU	Zhejiang University
CQUT	Chongqing University of Technology
LTU	Luleå University of Technology, Sweden
MIUN	Mid Sweden University
MARWIS	Mobile Advanced Road Weather Information Sensor
RCM411	Runway Condition Monitor 411
CNN	Convolutional meural networks
SVM	Support vector machines
ANN	Artiffical neural network
DCNN	Deep convolutional neural networks
ROI	Region of interest
BEV	Bird’s eye view
